# *Clostridioides (Clostridium) Difficile* in Food-Producing Animals, Horses and Household Pets: A Comprehensive Review

**DOI:** 10.3390/microorganisms7120667

**Published:** 2019-12-09

**Authors:** Melina Kachrimanidou, Eleni Tzika, George Filioussis

**Affiliations:** 1 First Department of Microbiology, Aristotle University of Thessaloniki, Medical School, 54124 Thessaloniki, Greece; 2 Farm Animal Clinic, School of Veterinary Medicine, Faculty of Health Sciences, Aristotle University of Thessaloniki, 54124 Thessaloniki, Greece; eltzika@vet.auth.gr; 3 Laboratory of Microbiology and Infectious Diseases, School of Veterinary Medicine, Faculty of Health Sciences, Aristotle University of Thessaloniki, 54124 Thessaloniki, Greece; georgefilious@vet.auth.gr

**Keywords:** *Clostridioides (Clostridium) difficile* infection, Zoonosis, food animals

## Abstract

*Clostridioides (Clostridium) difficile* is ubiquitous in the environment and is also considered as a bacterium of great importance in diarrhea-associated disease for humans and different animal species. Food animals and household pets are frequently found positive for toxigenic *C. difficile* without exposing clinical signs of infection. Humans and animals share common *C. difficile* ribotypes (RTs) suggesting potential zoonotic transmission. However, the role of animals for the development of human infection due to *C. difficile* remains unclear. One major public health issue is the existence of asymptomatic animals that carry and shed the bacterium to the environment, and infect individuals or populations, directly or through the food chain. *C. difficile* ribotype 078 is frequently isolated from food animals and household pets as well as from their environment. Nevertheless, direct evidence for the transmission of this particular ribotype from animals to humans has never been established. This review will summarize the current available data on epidemiology, clinical presentations, risk factors and laboratory diagnosis of *C. difficile* infection in food animals and household pets, outline potential prevention and control strategies, and also describe the current evidence towards a zoonotic potential of *C. difficile* infection.

## 1. Introduction

*Clostridioides (Clostridium) difficile* [1] *(C. difficile)* is a Gram-positive, anaerobic, spore-forming bacillus that colonizes the gastrointestinal tract of humans and animals [2,3]. The reclassification of *Clostridium difficile* to *Clostridioides difficile* in 2016 was based on phenotypic, chemotaxonomic and phylogenetic analyses [4]. The bacterium exists in two forms: the vegetative form highly sensitive to oxygen and the heat-stable spore form, which is able to survive a variety of harsh conditions. Although the first isolation of *C. difficile* was reported in 1935, it was not identified as the causative agent of pseudomembranous colitis and antibiotic associated diarrhea until four decades later [5]. Presently, *C. difficile* is one of the most common causal agents of nosocomial enteric infections in hospitals. Exotoxins A and B (TcdA and TcdB) are considered to be the major virulence factors associated with *C. difficile* infection [6,7,8,9,10].

Furthermore, *C. difficile* is recognized as an enteric pathogen in a variety of animal species, including food production animals (pigs, cattle, sheep, goats), horses and household pets (cats and dogs) [9,11,12,13]. Several studies have supported that animals can potentially act as vectors for the transmission of the bacterium to humans [14] via direct contact or via indirect transmission through raw food, or through contaminated water [15,16].

The incidence and the severity of *C. difficile* infection (CDI) have been significantly increased globally during the last 20 years [17]. Although the high-risk population consists of elderly hospitalized patients under antibiotic therapy, CDI cases in the community are also considerably increased. The emergence of new *C. difficile* rybotypes such as the 027 ribotype has changed the epidemiology of the disease. Another type of strain that is commonly isolated from patients with CDI in Europe is ribotype 078 [18].

Concerning veterinary medicine *C. difficile* has been widely recognized as the etiologic agent of enteritis in piglets [13,14,19]. In the Netherlands, ribotype 078 has been described as the dominant type in piglets with enteric infection [20]. Additionally, ribotype 078 is isolated in a high incidence from calves [21,22]. 

It is well established that humans and animals share common *C. difficile* ribotypes. Previous studies suggested that cows, pigs and chicken broilers could become possible sources of human CDI [21,22,23,24,25,26]. Findings on *C. difficile* in various animal species and an overlap in ribotypes (Davies, #9) suggest potential zoonotic transmission. However, the impact of animals for human CDI remains unclear. Many articles have summarized the changing epidemiology of CDI in humans, but the emerging presence of *C. difficile* in foods and animals has been infrequently addressed. However, there is clear evidence of human–animal transmission [27,28] as well as inter country spread of *C. difficile* via animal trading [29,30].

This review summarizes the current knowledge regarding the epidemiology, clinical presentations, risk factors, and laboratory diagnosis of CDI in animals. The available data about animals as vectors of CDI in humans is also presented. This is a narrative review and no formal inclusion or exclusion criteria are applied.

## 2. The Evolutionary History of *Clostridioides (Clostridium) difficile (C. difficile)* Detection in Animals and the Natural Environment

*C. difficile* infection has been described in various species of mammals including humans, pigs, horses, non-humans primates, rabbits, rats, domestic dogs, hamsters and domestic cats and is also found in the environment (soil, water, vegetables) [31,32,33,34,35]. 

*C. difficile* was first reported in mammals and birds during a biological survey in Antarctica and was also confirmed the asymptomatic carriage of the bacterium in stool samples from equidae and ruminants in Pakistan [36,37]. Later in 1979 the role of *C. difficile* as a causal agent of CDI infection in young gnotobiotic hares was documented [38,39]. In the next decade clinical reports of CDI in pigs [40] as well as the carriage of non-totoxigenic strains in goats [41] and cattle [42] were published. Ehrich et al., isolated for the first time, *C. difficile* from mature horses in a study of Potomac horse fever [43]. Furthermore, toxigenic strains of the bacterium have been associated with diarrhea in foals during sporadic cases as well as during severe outbreaks [44,45]. Meanwhile other studies focused on the isolation and confirmation of *C. difficile* from domestic pets, wild animals and ostriches [46,47]. Besides resent reports of asymptomatic carriage in white-tailed deer wild birds, barn swallows chimpanzees and zebras, rodents and feral pigs, the bacterium has also been isolated from Asian elephants and ocelots [47,48,49,50,51,52,53,54].

However, a number of studies described the presence of *C. difficile* in the natural environment of animal farms, water environment, and tropical soil [55,56,57]. These findings raised the concerns that domestic and wild animals could act as vectors for the spreading of *C. difficile* among humans.

Nowadays, the recent literature has presented many hypotheses regarding *C. difficile* transmission and specifically the risk of zoonotic *C. difficile* transmission [58]. Rupnik et al., reported that polymerase chain reaction (PCR)-ribotype 078 was the most prevalent type in pig, cattle and horse species worldwide, and also reported an increase in its prevalence in humans in different countries [59]. Further studies demonstrated that the most prevalent ribotypes in humans are also prevalent in various animal species from various geographic areas, suggesting the potential for global dissemination of specific *C. difficile* strains [60,61,62]. 

## 3. The Microorganism and Pathogenesis of *C. difficile* Infection (CDI)

*C. difficile* as a typical member of the genus *Clostridioides* is a Gram-positive, spore-forming, obligate anaerobe. In comparison to other anaerobes it grows slowly and it is often overgrown by other microorganisms in mixed cultures making the in vitro isolation very difficult. The genome size (Mb) of the bacterium ranges from 4.05 to 4.46, G+C content ranges from 28.4 to 29.2%, and CDS number ranges from 3485 to 4128 [63]. About 11% of its genome consists of transposons and prophages. These mobile genetic elements can be transferred horizontally between *C. difficile* strains acting as vectors of different genes including antibiotic-resistance genes [62]. Due to the numerous adaptations and the ability to tolerate bile salts *C. difficile* can survive in the intestine of humans and animals. Additionally, the microorganism can synthesize and tolerate 4-methylphenol (para-Cresol) an organic compound with bacteriostatic activity. Many intestinal microbes are sensitive to 4-methylphenol, enhancing the competitiveness of *C. difficile* against them [64].

The most common mode of transmission for the majority of species is fecal–oral route and/or the environment.

The organism is ingested either as the vegetative form or as spores and traverse the acidic stomach. *C. difficile* spores can easily overcome the stomach acidic barrier, continue to the intestine and colonize it under proper micro-environmental conditions [65]. *C. difficile* spores may survive for long periods, up to 5 months, on inanimate surfaces in the environment [66].

After colonization, the organism produces and releases the main virulence factors, the exotoxinsA (TcdA) and B (TcdB) [7,9] that disrupt epithelial integrity and cause intestinal inflammation, fluid, and mucous secretion, as well as damage to the intestinal mucosa [10]. TcdA is potent enterotoxic and possesses pro-inflammatory activities by interleukins(IL) IL-1, IL-6, IL-8, TNF-α by human monocytes as well as IL-6, and IL-8 by human intestinal epithelial cells [67]. However, only TcdB, which demonstrates cytotoxic effects, is recognised as the primary virulence factor [68]. These toxins encoded by genes TcdA and TcdB located on a 19.6 kb pathogenicity locus, the PaLoc. The PaLoc is present at identical chromosomal integration site in all toxigenic *C. difficile* strains. The binary toxin called binary *Clostridium difficile* ADP-ribosyltransferase toxin (CDT) was described from the *C. difficile* strain CD 196 by Popoff et al. [69] Binary toxin consists of a binding component (CDTb) and an enzymatic component (CDTa). CDT-encoding genes, *cdtA* and *cdtB*, are co-located on the chromosome outside the PaLoc on a locus CdtLoc [69,70]. Binary toxin is frequently present in *C. difficile* strains associated with increased CDI severity, and could, thus, be considered an additional virulence factor [70]. Similarly, pathogenesis of CDI is mediated by TcdA in domestic animals, since TcdB probably lacks binding capability in neonatal pigs and does not induce lesions in porcine intestinal explants [21,71]. However, with the appearance of the RT 027, several studies suggest that TcdB and not TcdA are essential for disease development. Moreover, the disruption of TcdB significantly reduces the virulence phenotype [68]. TcdA is a factor for fluid accumulation in animal models, whereas TcdB is not. However, TcdA is 1000 times less cytotoxic than TcdB [21,72]. Prior to this, TcdA was considered to be important for the development of diarrhea due to its enterotoxic nature. TcdB targets the epithelial cells after the mucosa is damaged by TcdA, since TcdB is not enterotoxic in experimental animal models [35,73]. However, there have been reports of clinical disease in humans by TcdA negative/TcdB positive strains [74,75], so TcdA is not essential for the development of the disease [35,73]. TcdA negative/TcdB positive strains were also found in pigs with diarrhea [76].

## 4. *C. Difficile* in Food-Producing Animals

### 4.1. C. Difficile in Food-Producing Animals: Swine

*C. difficile* has been widely described both in diarrheic and healthy pigs [22,77]. Newborn piglets are born having a sterile gastrointestinal tract which is being colonized by different kinds of bacteria within hours of life. This colonization is achieved via vaginal canal and sow’s perineum, feces, suckling, and via exposure to the environment within the first hours of life [33]. CDI is acquired from the surrounding environment and not by vertical transmission, since piglets born by caesarean section were culture negative [77]. A study in the US found higher *C. difficile* prevalence in cooler months (16.2%) than in warmer months (10.3%) in a vertically integrated pig farm [22]. Airborne dispersal of *C. difficile* spores has been also reported in a piggery [77]. Rodents and other vermin might play a role in *C. difficile* spreading. Intestinal content, skin and muscles were aseptically sampled for *C. difficile* from mice in a farm in the Netherlands. Additionally, dead insects and birds were also sampled. Skin of the mice was found having 51–66% culture prevalence compared to 8% for the gastrointestinal contents, whereas the predominant strain of *C. difficile* was RT078. Taken into consideration that the contamination rate of the body surfaces was higher than the gut, mice may spread *C. difficile* mechanically in the environment more likely than through the fecal route. It is also interesting that prevalence of *C. difficile* in dead sparrows was 66% and in various insects 56–100% [78]. *C. difficile* has also been isolated from urban rats, showing the possible role that vermin could play in dissemination of *C. difficile* in the environment [78]. Furthermore, the dosage of *C. difficile* is also an important factor for piglets to develop CDI, since increased prevalence and severity of microscopic lesions was associated with heavy bacterial loads [79]. The latest studies demonstrate that there is a specific microbiome that assists colonization resistance against CDI [80]. Kim et al., showed an alteration of microbiota balance in gnobiotic piglets using tigecycline (increased Proteobacteria and reduced Firmicutes), but it did not predispose piglets to CDI [81].

In piglets aged 1 to 7 days with CDI, the bacterium is associated with mesocolonic edema and pasty to watery yellowish feces in the large intestine [71]. Microscopic lesions include neutrophilic infiltration and variable amounts of fibrin, cellular and karyorrhectic debris. Inflammatory exudates are often related with multifocal coalescing ranges of disintegration and ulceration, known as volcano-like lesions. Histological lesions such as erosion, ulcerations and a neutrophilic infiltration are profoundly suggestive of malady, but not absolutely pathognomonic. Other symptoms are dyspnea, abdominal distension, scrotal edema, ascites and conspicuous edema at the ascending mesocolon, hydrothorax and kidney failure. Some piglets with CDI do not expose any clinical signs, although gross lesions of colitis are regularly seen at necropsy [14,19].

The prevalence of *C. difficile* in piglets between 1 and 2 weeks of age varies from 50% to nearly 100% in asymptomatic piglets [78,82], while there is a gradual decline as piglets grow older [78]. Although the morbidity of piglets at the same age with CDI can be as high as 100%, the average morbidity is on two third of litters and one third of individual piglets [14,20]. In contrast the mortality of the disease is usually low, but can be as high as 16% in severe outbreaks [71]. Piglets that have recovered from CDI have growth retardation resulting in 10% lower weaning weights on average [22]. Sporadic outbreaks of CDI in adult pigs are rare, although they can have significant consequences since adult pigs can also die [78]. Furthermore, deaths in per parturient sows (previously treated with enrofloxacin) have been also attributed to *C. difficile* [82]. Similarly, the presence of *C. difficile*-negative piglets has been described in healthy litters where 1.4% to 96% of the members carried the bacterium [78,82]. The incidence of CDI in adult pigs is low with sporadic outbreaks rare, the prevalence of *C. difficile* varies from 0% to 23% in finishing pigs, in pigs at slaughter houses and in per parturient sows [83,84]. This low incidence could be due to environmental conditions that prohibit the colonization of the bacterium before or after giving birth [78,85,86]. Weese et al., described the predominance of different PCR-ribotypes among farrowing sows and piglets suggesting that other sources than sows, probably environmental, could be responsible for the spreading of the bacterium among in newborn piglets [82]. In this regard, Keessen et al., detected spores of *C. difficile* in air samples of pig farms following relocation of piglets suggesting that the bacterium could be spread by aerosols [87]. Moreover on pig farms, *C. difficile* was detected in vermin leading to the proposal that they could act as vectors for bacteria transmission [88]. Squire and Riley, demonstrated that use of gloves and disinfection of a surface reduced the incidence and the mortality of CDI in piglets [77]. Despite the results of the previous study, the ways that pigs’ farms become infected by spores and viable cells of *C. difficile* still need to be clarified.

PCR ribotype 078 is the most common ribotype of *C. difficile* found in pigs [28,86,89]. However, there are studies that describe the predominance of different PCR-ribotypes in piglets and adult pigs in Europe. In Sweden, PCR ribotype 046 was the most common ribotype of *C. difficile* found in neonatal piglets, and also in humans [61,90]. Common ribotypes among piglets in Europe are RT066 in Slovenia [90,91], RT 126 in Germany [92], RT015, 023, 014 and 013 in the Netherlands [33,77,93].

### 4.2. Food-Producing Animals: Cattle

*C. difficile* was first detected in cattle as a contaminant of the intestine in the early 1980s. Generally, until recently *C. difficile* in ruminants was not well studied. Moreover enteritis of preweaning neonatal calf associated with a high mortality is the most common expression of cattle CDI (MLA 2003). Rodriguez-Palacios et al. reported a prevalence rate of 15% (20/134) in healthy calves and 7.6% (11/144) in calves with diarrhea [94]. In the same study 30.2% (16/53) of faces from healthy calves were toxin positive compared to 22.9% (58/253) of feces from diarrheic calves. In a subsequent study, any association between *C. difficile* colonization in calves and CDI was not confirmed [23]. Hammitt et al., were the first to describe the bacterium as a potential pathogen that causes enteritis in calves [23]. Previous studies failed to correlate the idiopathic enteritis in young calves with the colonization of the intestine by *C. difficile* [95].

It is well known that male calves are mainly used for veal production and are slaughtered at one month or at six months of age. Various studies established that the calves are colonized with *C. difficile* during the first day of their life. Therefore, the prevalence of the bacterium is higher in newborn calves and declines with the age demonstrated that veal calves less than 4 weeks of age were twice as likely to be colonized by *C. difficile* than those aged 36–45 days [96,97,98]. The high prevalence of *C. difficile* in calves could increase the risk of meat contamination at the abattoir. Moreover the diversity of *C. difficile* ribotypes in newborns is high and ribotypes like RTs 078, 126, 012, 045, 010, and 033 are usually isolated. This diversity of ribotypes diminishes as the veal calves grow older [96,97,98].

In addition to young age, putative risk factors for CDI in calves include antibiotic use and pure quality of colostrum. Metaphylaxis by the use of different antimicrobials appears to be a common practice in veal production globally [97,98]. It has clearly been shown that the use of antibiotics in veal production units is highly associated with high rate of *C. difficile* colonization in calves. The administration of colostrum could decrease the incidence of CDI, probably by providing passive immunity in the born calves by the definition of the role needs further investigation [23].

In contrast, the incidence rate of the bacterium in healthy or diarrheic adult bovines is lower than that observed in the calves. A possible explanation of this phenomenon is that the anaerobe colonizes and proliferates in the intestinal tract of calves easily since the normally protective commensal gut microbiota of young animals is less developed [23]. Finally the incidence of *C. difficile* in dairy cows also seems to be very low. A study testing for *C. difficile* was performed on 118 dairy operations across the 17 participating states in USA. Overall, 1858 fecal samples from dairy cows were tested for the presence of *C. difficile* which was isolated from 29 samples (1.6 percent) [61].

The predominant ribotype in calves is 078 [35,61]. Moreover, there are studies that report the predominance of different ribotypes in calves in Europe such as 033, 077 and 038 in Slovenia [91,99,100]; 126, 045, 033, 012, 029 and 015 in Belgium [99]; 137, 033, 066, 003 and 070 in Switzerland [101]; 033, 078 and 045 in Germany and 012 in the Netherlands [102,103].

### 4.3. Food-Producing Animals: Poultry

Clinical manifestation of CDI infection in poultry is necrotizing enteritis [104,105]. Clinical signs include acute onset of diarrhea and a subsequent rapid progression to death. Infected birds usually die within 3 days of symptom onset. The mortality rates of poultry with CDI are usually very high [47]. The characteristic gross lesions of the infected birds are disseminated multifocal hemorrhages in ceca and colon and watery feces in the small intestine. Finally the characteristic microscopic lesions are edema in cecal wall and colon as well as severe fibrinonecrotic typhlocolitis [47].

The prevalence of *C. difficile* in poultry has been recently documented. Studies undertaken in Africa showed that up to 30% of free-range chickens carried toxigenic *C. difficile* strains that also were resistant to antibiotics used in human medicine [105,106]. These results indicated that poultry meat can be considered as a vector for the transmission of *C. difficile* from poultry to humans. Meanwhile, the prevalence of *C. difficile* in some Asian and Latin American countries found to be very high and was correlated to the high incidence of CDI infections in humans [107,108,109,110,111]. In another study conducted in Egypt *C. difficile* prevalence was 11.5% (12 of 104) in poultry, 14% (7 of 50) for healthy and 9.3% (5 of 54) for diseased poultry, respectively [112]. Moreover similar studies also documented the prevalence of *C. difficile* in poultry in European Union as well as in the USA. Previous studies have shown that in European countries, the isolation rate of the pathogen is generally lower than 5% in poultry [113,114]. In contrast higher incidence levels, up to 42%, were determined, whereas the reported prevalence rates in North America and Canada were ranged from the lower 2% to the higher up to 44% [114,115].

The age effect is also described in poultry, where a high prevalence of 62% was found in young poultry which decreased with increasing age [58]. The prevalence in poultry varies between 1.6% and 29% [26,105,106]. However, the highest prevalence recorded was found in a layer farm in Slovenia (62.3%) with a high genotypic diversity of the isolates, most of them non-toxigenic [24] with the predominance of ribotype RT023. High genetic diversity but low prevalence in poultry was observed in India (prevalence = 14%, RTs = 13), Austria (prevalenc e = 5%, RTs = 3) and the Netherlands (prevalence = 5.8%, RTs = 5) [26,93,112,116].

The frequent isolation of ribotypes which are also found in humans constitutes a substantial overlap and makes poultry meat a potential source for *C. difficile* infection in humans.

### 4.4. Food-Producing Animals: Sheep and Goats

Sheep and goat have mainly been reported as asymptomatic carriers of *C. difficile*, with a prevalence varying between 0.6% and 10.1%. The low incidence of the bacterium in small ruminants may be attributed to the limited use of antibiotics in these species [76]. However, the direct relationship between the use of antibiotics and the incidence of asymptomatic carriage of *C. difficile* or CDI are not directly related [100]. The rate of *C. difficile* in sheep and goats seems to decrease with age. In a recent study conducted in Slovenia investigated the diversity of *C. difficile* in two groups of 109 healthy goats and 105 healthy sheep belonged in different ages. The organism was detected in 9.2% of the goats and 5.7% of the sheep enrolled in the study. Only young animals were positive. The recovered strains were categorized in 010, 014/020, 045, 056, SLO 061, and SLO PCR-ribotypes [89]. The colonization of healthy sheep and goats with the bacterium proliferate the animal-to-animal transmission as well as the zoonotic transmission.

## 5. *C. difficile* in Horses

Horses of any age can develop CDI either as outbreaks or as sporadic cases [82]. The main sources of the bacterium are infected animals that shed the bacterium to the environment [116]. Transmission occurs by ingestion of bacterial spores in which the majority of disinfectants are ineffective [117]. There are many reports on *C. difficile* infections in foals, although the clinical manifestation differs from that in adult horses. A main difference is that CDI in foals can occur without prior use of antibiotics [44]. Clinical manifestation of CDI in foals includes enterocolitis characterized by acute and watery diarrhea. The symptoms could arise shortly after birth and if not treated, the mortality rate is very high. Lesions of the disease are focused on the duodenum, ileum and jejunum of the foal [118]. In a recent study, the effect of probiotics on diarrheic foals during the first six months of their life was evaluated. Although the foals received probiotics for 3 weeks no observable effect on them was reported.

Moreover foals can also be asymptomatic carriers of the bacterium. Båverud et al., reported an asymptomatic carriage of *C. difficile* of 44% in foals that were under treatment with erythromycin and rifampicin, and 15% with a combination of trimethoprim/sulfonamides and penicillin [119]. Moreover, 28.5% of foals that were identified as asymptomatic carriers were younger than two weeks of age. Asymptomatic carriage of *C. difficile* in foals younger than two weeks was reported in many studies making the association between positive fecal samples and CDI questionable [43,44]. Foal-mare pairs can harbor *C. difficile* without exposing any clinical signs and contaminate the environment [120]. The prevalence of *C. difficile* in very young foals, aged less than 14 days was 29% and decreases to 0–1% in horses older than 14 days [114]. A high prevalence of *C. difficile* is reported in horses with diarrhea varying from 12.7% to 42% [114].

In adult horses, CDI is characterized by non-typical signs like dehydration, anorexia, pyrexia, tachycardia, and tachypnea. Additionally clinical signs from the intestine like tympanic abdominal distension and diarrhea often associated with colic may be present [121]. In some cases, sudden death may occur even before the onset of any clinical sign. Lesions of the disease are focused on the cecum and colon of the adult horse [118]. Schoster et al., suggested the asymptomatic colonization of the intestine of adult horses and the shedding of *C. difficile* to the environment [122]. In hospitalized asymptomatic adult horses the prevalence of *C. difficile* ranged from 4.8% to 11% [120,123].

A great diversity of *C. difficile* ribotypes has been reported for horses [27]. Predominant ribotypes in horses 015, 033, 078 and 001 are also well-known in humans [117]. A recent study conducted in the Czech Republic, reports a great diversity of *C. difficile* ribotypes in horses (7 ribotypes: 033; 081; 009; 003; 010; 012; 039, including toxigenic and non-toxigenic *C. difficile* strains while ribotype 033 predominates [124]. Reports from Slovenia, Italy, the Netherlands and Belgium demonstrated *C. difficile* carriage rates from 3.7% to 33.3% with a remarkably high diversity of detected ribotypes, just ribotype 014 was detected in three of the five studies [89,123,125].

## 6. *C. Difficile* in Household Pets: Dogs and Cats

The importance of dogs and cats as common vectors for the transmission of *C. difficile* in owners was referred to by Rodriguez-Palacios et al. [17]. Too many household pets are considered as members of the family and have an access in the living place. According to a study it was indicated that 10% of household dogs were asymptomatic colonizers of the bacterium and spores contaminated the 31% of the households [17]. The young, elderly, as well as immunocompromised isolates of pet origin were different from isolates of human origin [32]. Therefore, the potential circulation of *C. difficile* strains among dogs and humans is still unclear. Stone et al., reported the isolation of *C. difficile* in 17% of the canine fecal samples of asymptomatic dogs [126]. Interestingly 10% of the isolates were toxigenic strains that cause CDI in humans. Sequencing analyses revealed similarities among dog and human genotypes. These results suggest that household pets can be potential sources of community acquired *C. difficile* infections in humans. According to Rodriguez-Palacios et al., immunocompromised owners were potential shedders of the bacterium to their dogs [17]. Moreover, the household pets were at risk to develop CDI. Dogs that visited human hospitals were demonstrated to be at high risk of becoming asymptomatic carriers of *C. difficile* [126]. The risk proved to be very high in the case that the dogs were under therapy with antibiotics [127]. The ribotype 027, a typical human epidemic strain, was isolated from a healthy dog that was visiting patients in a hospital on a weekly basis [126].

CDI in pets is attributed to community-associated strains rather than to strains acquired from other infected animals [128]. However colonization of pets with strains from veterinary hospitals was also reported and the administration of antibiotics has been considered as a risk factor [129]. Clinical manifestation of CDI ranges from mild self-limiting diarrhea to severe and fatal diarrhea. Many studies have associated the isolation of the bacterium from faces with diarrhea in household pets [129,130,131]. A high prevalence of non-toxigenic strains has been described in dogs and cats underlying the importance of the detection of toxins when diagnosing CDI [129]. There is also significant association between toxins and diarrhea in dogs [129,132,133]. Dogs can also be asymptomatic carriers of *C. difficile* strains belonging to human epidemic PCR-ribotypes [89,129]. The higher incidence of carriage is reported in young dogs. The available information about CDI in cats is limited. In hospitalized cats, the colonization rate has ranged from 9.4% to 31%. Additionally, the colonization rates of *C. difficile* in healthy dogs and cats in the community range from 1.4% to 21% [131]. Madewell et al., suggested that the clinical environment can be a source for the contamination of cats with *C. difficile* [134]. Moreover, raw and processed food has been identified as vectors for the contamination of household pets with the bacterium [135]. Given the close contact between household pets and humans it is, therefore, desirable to screen the animals which may come into contact with people at risk, such as immunocompromised individuals and the elderly, in order to prevent the occurrence of CDI.

Rabold et al. reported the occurrence of eight different *C. difficile* ribotypes (RTs) 001, 009, 010, 014, 014, 027, 039 and 078 in dogs and cats [136]. Even though the predominant RT was 014, RTs 027 and 078, ribotypes that are often described as highly pathogenic for humans, here they originated from dogs [136].

## 7. Epidemiology—*C. difficile* Polymerase Chain Reaction (PCR) Ribotypes in Animals in Europe

The epidemiology of *C. difficile* has been studied using a variety of typing methods, including whole-genomes sequence analysis (WGS), which has also allowed the comparison of animal, food and human isolates [137]. Lemee et al., investigated the genetic relationships and population structures of *C. difficile* isolates from various hosts and geographic sources, including human, dog, horse, cow and rabbit stools [138]. Results demonstrated that animal isolates did not cluster separately from human isolates, but instead were intermixed with human lineages. Epidemiological studies reported the PCR-ribotype 078 as the predominant type in pig, cattle and horse species worldwide, and also reported an increase in its prevalence in humans [59,93]. However, a high degree of similarity between pig and animal *C. difficile* PCR-ribotype 078 strains was observed suggesting a common origin [24]. Janezic et al., reported that the most prevalent *C. difficile* ribotypes in humans are also prevalent in animals from different geographic locations, suggesting the potential for the global spread of some strains [139].

A great variety of *C. difficile* PCR ribotypes has been reported in different farm animals in Europe. PCR ribotype 078 is described as the dominant type in swine throughout Europe [12,24,61,90]. There are also other PCR ribotypes isolated from pig farms such as RT 002, 014, 015 and 023; however, they have only been reported in specific studies [61,86,89,90]. In cattle, PCR ribotype 078 has also been commonly detected in different European countries [12,26,61,140] Calves were mostly colonized already upon arrival to the farm and ribotypes 078 and 126 were persisting from the beginning to the last stages of the production cycle. There is also another PCR ribotype, 033, that seems to be cattle-associated and has been reported in Belgium, Germany, Switzerland and Slovenia. Other frequently isolated PCR ribotypes in cattle were 012 and 002 in Belgium, The Netherlands and Slovenia [12,26,90]. As far as the household pets are concerned, the most frequently reported ribotypes across Europe are 039 or 039/2, 014 or 014/020, 010 in cats [61,90,136] and 010, 014/020, 056, 078, 039, 009, 012, 106 in dogs [51,61,90,129,132,136].

## 8. Diagnosis

The diagnosis of CDI is based on clinical investigation (presence of symptoms and predisposing factors) and laboratory confirmation. Major step for the diagnosis of porcine CDI is the detection of TcdA and TcdB in feces or colonic content. The reference method is measurement of neutralizable cytotoxicity in monolayers of Chinese hamster ovary or other cells. However commercially available enzyme immunoassays are widely used [141]. There are different laboratory methods of diagnosing CDI, but still is unclear which is optimal [142]. The most commonly used laboratory tests are:

### 8.1. C. Difficile Isolation—Anaerobic Stool Culture

Isolation of *C. difficile* can be performed either by culture on agar media, enrichment for spore formation, or by the CCCN (cell culture cytotoxin neutralization) test. Culture methods for *C. difficile* are considered sensitive but not specific for diagnosis because non-toxinogenic strains, which are considered non-pathogenic, but can be found in faeces of both symptomatic and asymptomatic animals [143]. One of the most widely used selective agar media for *C. difficile* isolation from stool is pre-reduced cycloserine-cefoxitin-fructose agar [144], which may be supplemented with taurocholate to enhance spore germination [145]. This medium is often used in conjunction with an enrichment broth, such as cycloserine-cefoxitin-mannitol broth with taurocholate, lysozyme, and cysteine, to enhance the isolation of *C. difficile*.

Fecal samples are cultured on selective agar, cycloserine-cefoxitin-fructose agar, directly and also after an enrichment culture. Fecal samples are resuspended in 1–2 mL of 0.85% NaCl and 0.5 mL is transferred into 5 mL of enrichment broth. After 2 days of incubation, 0.5 mL of enrichment culture are mixed with an equal amount of ethanol, incubated for 30 min at room temperature, and then centrifuged at 10,000× *g* for 5 min. The supernatant fluid is discarded and the pellet is inoculated onto a selective medium cycloserine-cefoxitin-fructose agar. Plates are inspected after 3 days of incubation under anaerobic conditions at 37 °C. *C. difficile* colonies have a ground-glass appearance and smell of *para*-cresol (similar to a horse barn) and fluorescence under ultraviolet (UV) illumination (yellow-green) [146,147]. 

### 8.2. Characterization of C. Difficile Strains

- **Detection of PaLoc genes and CDT locus Genes by** Polymerase Chain Reaction

Ligonucleotide primers are used to detect the *tcdA* (encoding toxin A), *tcd*B (encoding toxin B), and *tcd*C (encoding a negative regulator of toxin A and B) sequences found within the pathogenicity locus operon (PaLoc) [148]. Oligonucleotide primers are also used to detect the *cdtA*/*cdtB* genes (encoding *binary toxin*).

- **Multilocus sequence typing (MLST)**

Multilocus sequence typing (MLST) is a nucleotide sequence-based characterization of allelic polymorphism of housekeeping genes. Allelic profiles allow the definition of different sequence types (STs). MLST provides sequence data that can be generated from various laboratories and should be shared in a common web database. A combination of MLST and MLVA (Multiple-locus variable-number of tandem-repeats analysis) provide phylogenetic information that will be valuable for investigations of *C. difficile* population [138,148].

- **PCR ribotyping**

PCR ribotyping exploits differences in the spacer regions of 16S and 23S ribosomal RNA, using specific primers that encode these RNA regions [149,150]. The gel electrophoresis reveals a few DNA bands that are referred to as ribotypes. This molecular typing technique is more commonly used throughout Europe.

- **Toxinotyping**

*C. difficile* shows considerable variability in the PaLoc region, encoding two main virulence factors, toxins TcdA and TcdB. Strains with changes in PaLoc are defined as variant toxinotypes, and currently 31 groups (I to XXXI) are recognized. Toxinotype 0 strains contain a PaLoc identical to the reference laboratory strain VPI 10463 to which all changes in PaLoc are compared. Toxinotyping is a Restriction Fragment Length Polymorphism (RFLP)-PCR based method for differentiating *C. difficile* strains according to changes in their toxin genes when compared to the reference strain VPI 10463 [151].

- **Whole-genome sequencing (WGS)**

Whole genome sequencing (WGS) has recently been used to study the epidemiology of CDI and the genetics of *C. difficile* [2]. Such studies investigate the evolutionary relatedness of *C. difficile* strains isolated from humans and animals, in order to reveal identical or nearly identical *C. difficile* clones, supporting the hypothesis of interspecies transmission between animals and humans [152].

A rapid and accurate diagnostic approach for CDI is a key step for the prevention and control of CDI. The idea of performing a 2- or 3-step laboratory algorithms has been proposed since 2006, aiming to improve the performance of the *C. difficile* test to optimize their specificity and sensitivity, improving CDI diagnosis [153].

## 9. Control of *C. Difficile*—Prevention

Low temperatures (4–5 °C), high humidity and quantity of inoculum are potential causes of *C. difficile* spores persistence in the environment [154]. Since *C. difficile* produce spores that are resistant to most disinfectants, heat and ultraviolet light, it is extremely difficult to eliminate them from the farrowing environment. Therefore, that should be an important consideration for veterinarians managing such infections in neonates [14]. Some epidemic strains of *C. difficile* may have a higher sporulation capacity than non-epidemic strains and thus they persist in the environment longer [155]. However, Robinson et al., did not confirm the difference in sporulation capacity between hypervirulent and non-hypervirulent strains [156].

As observed in mouse and hamster models, antibodies against TcdA and TcdB prevent toxin binding, and consequently reduce secretion, inflammation, and clinical disease. Thus, immunoprophylaxis of CDI in domestic animals should probably be antitoxic. Antibodies against TcdA and TcdB prevent toxin binding in mouse and hamster models, eliminating secretion, inflammation and clinical disease [157]. In vitro antimicrobial susceptibility testing reported tylosin as an effective tool for the treatment of CDI in piglets. Concerning other antibiotics, erythromycin and tetracycline can be also useful for treating piglets and tiamulin or virginiamycin for reducing *C. difficile* levels in adult pigs. There are also studies suggesting potential and variable benefits of probiotics against CDI in animals [158,159]. Live non-toxigenic strains of *C. difficile* and non-pathogenic yeast, *Saccharomyces boulardii*, can be also used [159,160]. It has been reported that formalin inactivated *C. difficile* non-toxigenic strains and toxoids can give parenteral and mucosal immunization showing high serum concentrations of toxin neutralizing antibodies. Kink et al., reported that antibodies to recombinant toxins A and B can be used as a treatment [157].

Research on other species suggested particular antibiotics such as metronidazole and vancomycin as effective in horses but those two are not approved for use in food animals [143,161]. Bacitracin methylene can be used for preventing but also for treating enteritis due to *C. difficile*. Oral administration of *C. sordellii* antitoxin is used to prevent enteritis in hamsters [162].

Effective control can be exercised by reducing the environmental burden of *C. difficile* through a “One Health” approach, along with boosting the host defense against the virulent enteric pathogen. The “One Health” concept connects the health of humans to the health of animals and their shared environments, representing a relevant framework for better understanding of the emergence and spread of CDI in humans and animals and also the inter-species clonal transmission. To that end, it has been proposed to administer vaccines against *C. difficile* to reduce carriage in animals [16,163]. Other initiatives including composting of biosolids or thermophilic sludge digestion have proven to be effective interventions to reduce the carriage of *C. difficile* in biosolids [83]. Increasing the resistance of the host to CDI through protective microbiota and immune effectiveness would also be an effective protective approach. Disrupted interactions between the microbiome and host immune system due to dysbiosis inhibit other host-encoded mechanisms to limit *C. difficile* infection and disease.

## 10. Future Perspectives

Since the last decade, *Clostridioides difficile* has remained a major cause of attention in hospitals and also an important topic for research worldwide. Comparisons of strains have revealed that animals and humans can be colonized with identical *C. difficile* clones or strains that cluster in the same lineage. Therefore, it is suggested that *C. difficile* should be considered as a zoonotic pathogen and the interspecies transmission between animals and humans and also the existence of a common contamination source is possible with animals as a reservoir for humans. These findings highlight the importance of a comprehensive One Health perspective in monitoring and controlling *C. difficile* infection. While many questions remain unanswered, increasing availability and affordability of next generation typing techniques is likely to advance our understanding of transmission of *C. difficile* in the years to come, allowing comparison of transmission events between different environmental niches, humans and animals that will definitely provide new insights in *C. difficile* biology and epidemiology and will aid in therapeutic interventions.
